# Evaluating neighborhood structures for modeling intercity diffusion of large-scale dengue epidemics

**DOI:** 10.1186/s12942-018-0131-2

**Published:** 2018-05-03

**Authors:** Tzai-Hung Wen, Ching-Shun Hsu, Ming-Che Hu

**Affiliations:** 1 Department of Geography, National Taiwan University, No. 1, Sec. 4, Roosevelt Road, Taipei City, 10617 Taiwan; 2 Department of Bioenvironmental Systems Engineering, National Taiwan University, No. 1, Sec. 4, Roosevelt Road, Taipei City, 10617 Taiwan

**Keywords:** Dengue, Epidemic diffusion, Spatial regression, Human mobility, Taiwan

## Abstract

**Background:**

Dengue fever is a vector-borne infectious disease that is transmitted by contact between vector mosquitoes and susceptible hosts. The literature has addressed the issue on quantifying the effect of individual mobility on dengue transmission. However, there are methodological concerns in the spatial regression model configuration for examining the effect of intercity-scale human mobility on dengue diffusion. The purposes of the study are to investigate the influence of neighborhood structures on intercity epidemic progression from pre-epidemic to epidemic periods and to compare definitions of different neighborhood structures for interpreting the spread of dengue epidemics.

**Methods:**

We proposed a framework for assessing the effect of model configurations on dengue incidence in 2014 and 2015, which were the most severe outbreaks in 70 years in Taiwan. Compared with the conventional model configuration in spatial regression analysis, our proposed model used a radiation model, which reflects population flow between townships, as a spatial weight to capture the structure of human mobility.

**Results:**

The results of our model demonstrate better model fitting performance, indicating that the structure of human mobility has better explanatory power in dengue diffusion than the geometric structure of administration boundaries and geographic distance between centroids of cities. We also identified spatial–temporal hierarchy of dengue diffusion: dengue incidence would be influenced by its immediate neighboring townships during pre-epidemic and epidemic periods, and also with more distant neighbors (based on mobility) in pre-epidemic periods.

**Conclusions:**

Our findings suggest that the structure of population mobility could more reasonably capture urban-to-urban interactions, which implies that the hub cities could be a “bridge” for large-scale transmission and make townships that immediately connect to hub cities more vulnerable to dengue epidemics.

**Electronic supplementary material:**

The online version of this article (10.1186/s12942-018-0131-2) contains supplementary material, which is available to authorized users.

## Background

Dengue fever is a vector-borne infectious disease that is transmitted by contact between vector mosquitoes and susceptible hosts [1]. Since the 1970s, dengue fever has been gradually spreading throughout tropical and sub-tropical countries, and its transmission involves interactions among carriers, mosquitoes, and healthy humans. More than 125 countries are impacted by the disease, and it is an increasingly serious threat to global public health due to climate change. Previous studies showed meteorological and social–economic risk factors that facilitate the disease transmission, including temperature, rainfall, population density, demographic composition, urbanized levels and more [2–4]. The Fifth Assessment Report (AR5) of the Intergovernmental Panel on Climate Change (IPCC) also confirmed that global warming would create more suitable habitats for vector mosquitoes in sub-tropical regions and speed up the geographic expansion of dengue epidemic areas due to global mobility including to some high-latitude countries such as France and Japan [5–12].

Due to the limited flight range of mosquitoes [13], it is impossible for the virus to be transmitted to distant areas by dengue vectors. Population movement across countries by air traffic is the major driver of the international spread of the disease [12–17]. Via air travel, disease importation from dengue-endemic countries is a trigger point for initiating indigenous epidemics in some dengue-epidemic countries or regions, such as Tokyo, Japan; south-east France; and southern Taiwan [14, 18]. Routine mobility behaviors, such as daily commutes, are also drivers of large-scale intercity transmission [19]. Therefore, understanding the spatial structure of population mobility is crucial for assessing the possible mechanisms of dengue diffusion and identifying the geographic characteristics of high-risk areas [20].

Recent studies on assessing the influence of human mobility on dengue transmission can be categorized into three approaches. The first is to construct simulation or statistical models that incorporate human mobility as the mechanism of dengue diffusion [21]. For example, Barmak et al. [22] showed that the long-distance mobility pattern is an efficient pathway for dengue transmission. Another study used survival analysis to show that daily routine commuters facilitate the large-scale spatial–temporal diffusion of the epidemic in a city [19]. The second perspective is to collect human mobility or behavior data to analyze the spread of dengue. Stoddard et al. [15] showed that small-scale mobility behavior among households also played an important role in dengue epidemics in Iquitos, Peru. Wesolowski et al. [16] used the Call Detail Records (CDRs) from mobile phones to analyze the spatial behaviors of humans in Pakistan for predicting diffusion of dengue in time and space. Airline traffic data are also available for studying the international spread of dengue epidemics and assessing the disease importation risk from dengue-endemic countries [23, 24]. The third perspective is to analyze geometric structures of geography to measure geospatial similarity or neighborhoods as a surrogate for human mobility. Spatial regression modeling is the major approach for measuring the neighborhood effects on dengue risk after controlling for environmental factors [4, 25].

The above studies showed that human mobility could be the main risk factor for dengue transmission on both the regional and global scales. However, methodological concerns remain for examining the effect of intercity human mobility. First, spatial settings in the regression model often examine the geometric relationships of geography as a surrogate for spatial interactions and human interactions. For example, the weights of spatial contiguity can be defined as administration boundaries with common borders and points [26] or areas based on k-nearest neighbors within a specific distance [25]. These definitions may simplify the complex interactions of humans because the geometry of spatial contiguity cannot comprehensively reflect these human interactions due to topographical or social–economic barriers across the study area [27]. Moreover, the spatial heterogeneity of human interactions or mobility may not be captured by the geometry of the boundaries alone. In addition, it is difficult to differentiate the effect of urban-to-urban or rural-to-rural mobility on epidemic diffusion if these areas share similar geometric structures. Some studies suggest that the use of real population flow could act as a spatial weight that captures more realistic spatial interactions [27, 28]. Therefore, approaches that use Global Positioning System (GPS) logs, cell phone records or geotags from social media for tracking moving trajectories of individuals have become emerging methods for studying human mobility and dengue risk [29, 30]. However, massive cell phone data provided by telecommunication companies are often difficult to access in the research community. Tracking collective behaviors from cell phone data may also violate location privacy, and this approach could be controversial in most developed countries. Due to these concerns, mathematical models, such as gravity, spatial interaction or radiation models, are used to estimate population flow across cities. Spatial models have become widely used approaches to study the geography of human mobility and disease transmission [29, 31, 32]. Among these models, the radiation model is a parameter-free algorithm that is robust in estimating the flow of intercity human mobility [32].

To clarify the role of intercity human mobility, we used the radiation model to capture the structure of spatial mobility as a possible mechanism. We examined the effect of neighborhood structures on the spatial–temporal spread of dengue epidemics in southern Taiwan from 2014 to 2015, the most severe outbreaks over the course of 70 years in Taiwan, and identified the common social-demographic features in these high-epidemic regions. By profiling the neighborhood effects on the spatial–temporal structures of disease spread, we proposed a study framework for interpreting possible pathways of intercity diffusion of dengue epidemics. The purposes of the study are (1) to investigate the influence of neighborhood structures on epidemic progression from the pre-epidemic to epidemic periods and (2) to compare the definitions of different neighborhood structures for interpreting the spread of dengue epidemics.

## Data and methods

### Study area

**Fig. 1:**
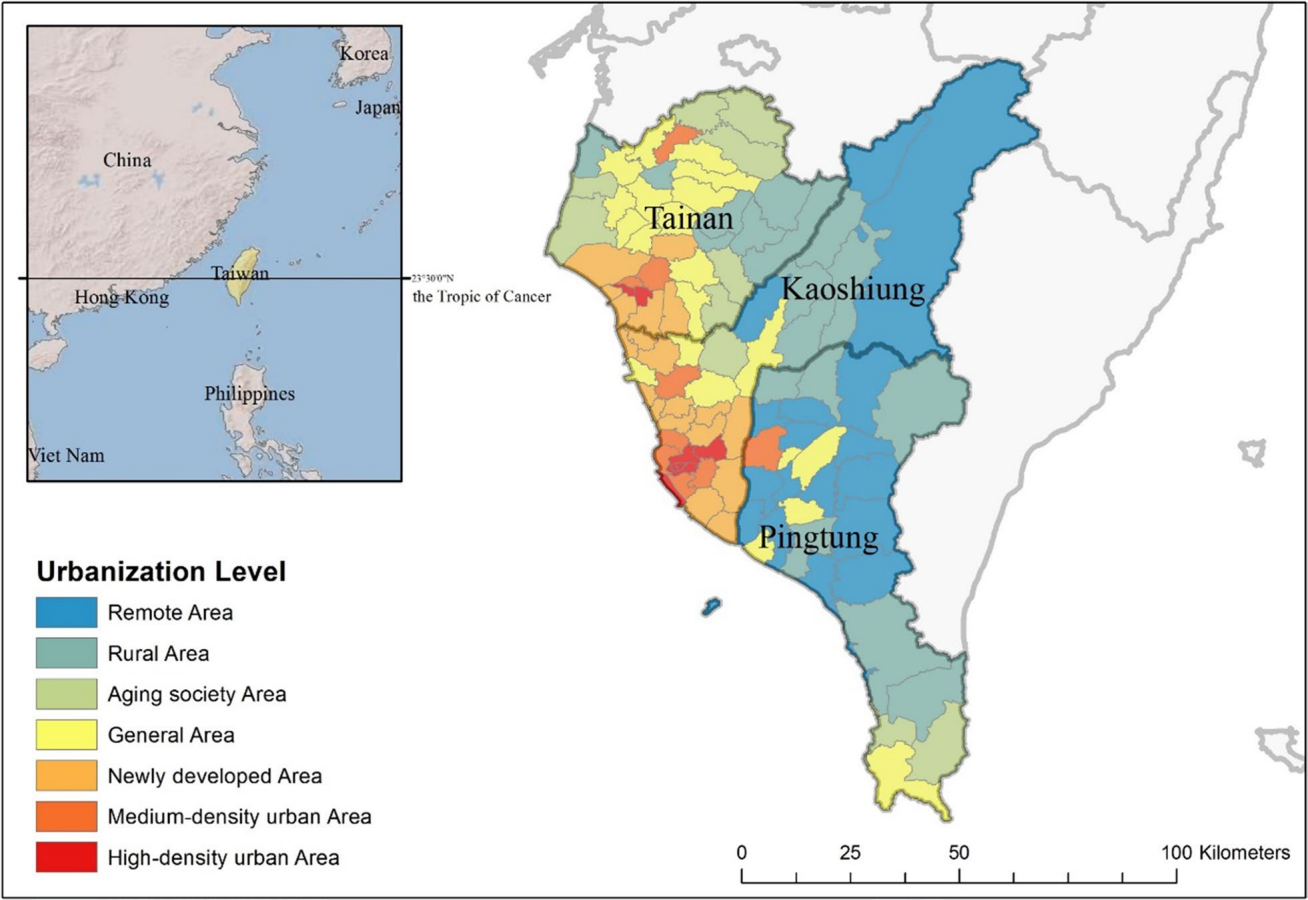
Urbanization levels in southern Taiwan

Southeast Asia is one of the major dengue-endemic regions in the world [33–36]. Taiwan is located in the border region of Southeast and East Asia. Southern Taiwan, which is passed through by the Tropic of Cancer, has a tropical monsoon climate; it is dry in the winter and hot and wet in the summer and autumn. The population has grown quickly, reaching 5.5 million in 2014. With an average of 683 persons/km^2^, metropolitan areas of southern Taiwan have become one of the most densely populated areas in the world. Due to its climatic and demographic characteristics, the region is a severe dengue-epidemic region of Taiwan, which annually covers more than 85% of the total confirmed dengue cases in Taiwan. Therefore, this region, including Tainan and Kaohsiung Cities and Pin-tung County, was used as the study area. A township was used as the unit for analysis, which is the basic unit for regional master planning and national policy implementation. Our study analyzed the dengue incidence and profiled the social–economic structures of spatial diffusion in 108 townships from 2014 to 2015. To differentiate between the social–economic statuses of each township, we categorized the urbanization levels into seven types, including highly or middle-developed, emerging, general, aging, rural, and non-developed areas as shown in Fig. 1. These types were determined by socio-demographic variables, including population density, population ratio of people with college or above educational levels, population ratio of elder people over 65 years old, population ratio of people of agriculture workers, and the number of physicians per 100,000 people from Taiwan census database [37].

### Dengue epidemics in study area

**Fig. 2:**
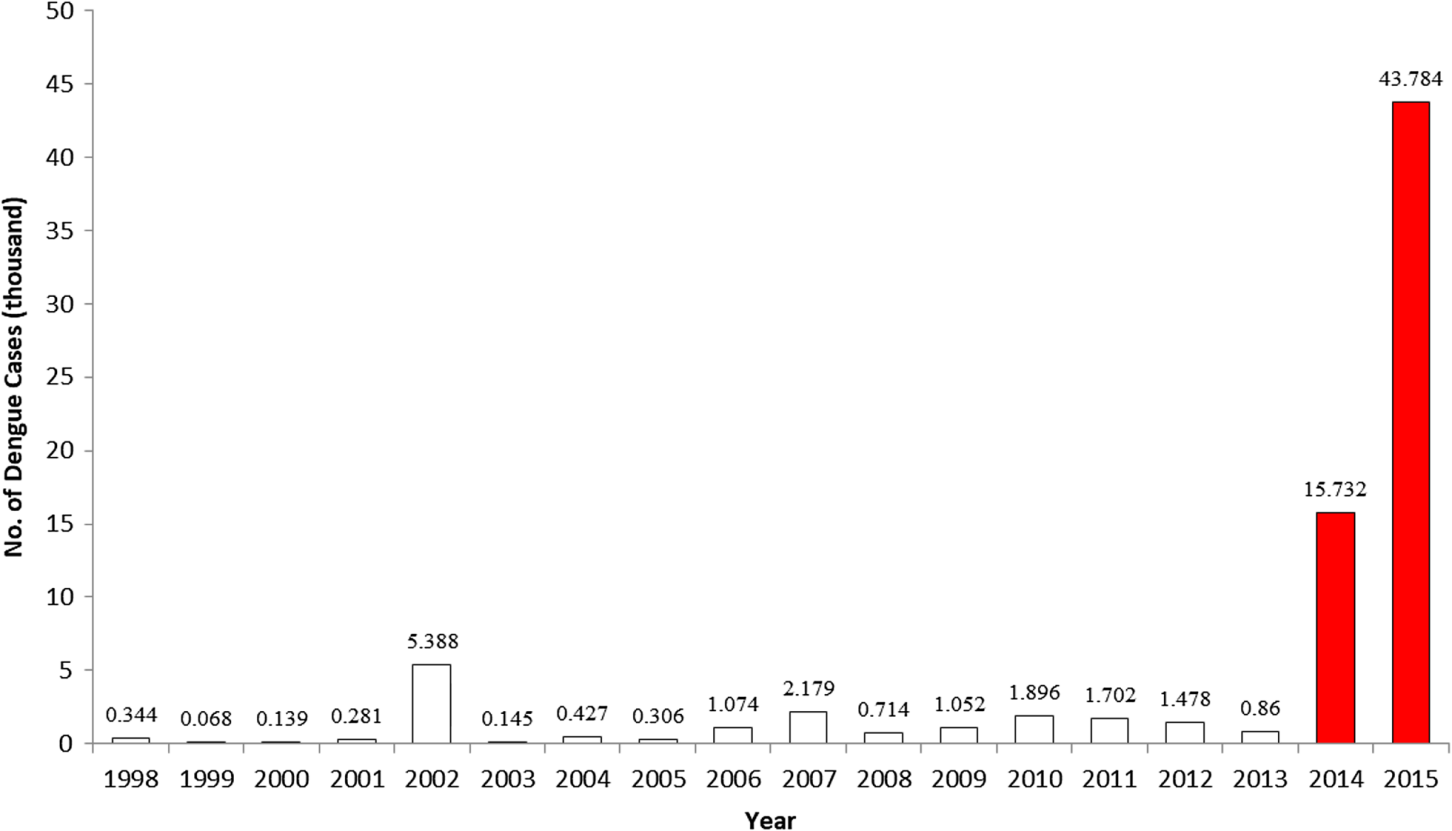
Number of dengue cases in Taiwan (1998–2015)

**Fig. 3:**
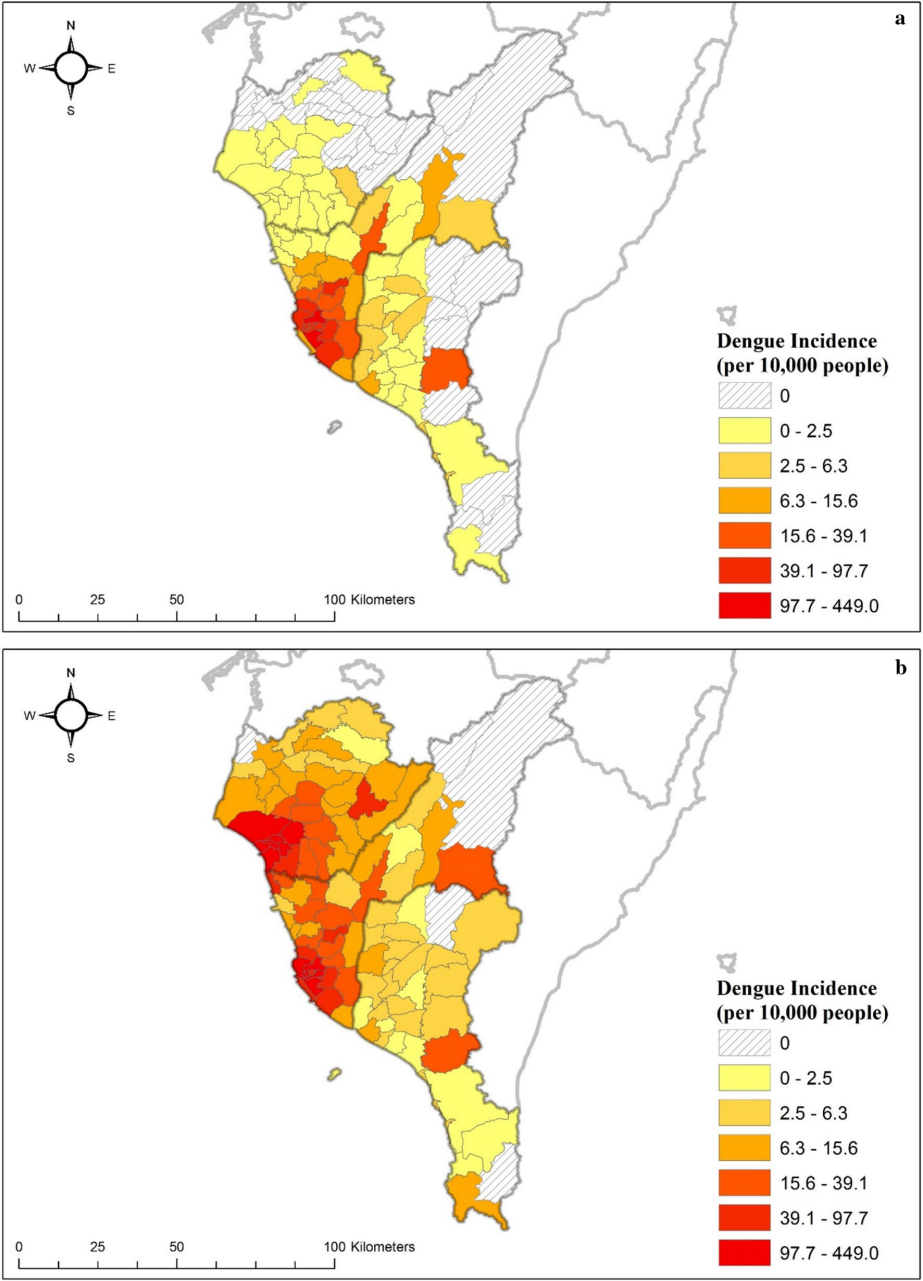
Spatial distributions of dengue incidence in **a** 2014 and **b** 2015

Dengue fever is a notifiable infectious disease in Taiwan. The dengue surveillance data from the Taiwan Centers for Disease Control (Taiwan CDC) are based on institutional reporting and border surveillance. The confirmed dengue cases reported by the Taiwan CDC are laboratory-positive dengue cases, which indicates a suspected dengue case with anti-dengue IgM seroconversion or single anti-dengue IgM positivity or a case with dengue virus identification through RT-PCR [38]. Their residences of cases were also aggregated as counts in townships for public announcement. Figure 2 shows the temporal trend of dengue epidemics from 1998 to 2015 in Taiwan. The figure indicates that, in the last 2 years, the number of confirmed cases reached 15,732 and 43,784, and 233 people dead, respectively, which are the most severe outbreaks over the course of 70 years in Taiwan. Most high-epidemic areas were concentrated in Tainan and Kaohsiung Cities (Fig. 3). Moreover, the southern Taiwan is located in the border of tropical and sub-tropical climatic zones (Fig. 1). Therefore, the dengue epidemics in Taiwan can be regarded as one of dengue sentinel indicators in Southeast and East Asia, which monitors geographic expansion of dengue epidemics to middle or high-latitude countries.

**Fig. 4:**
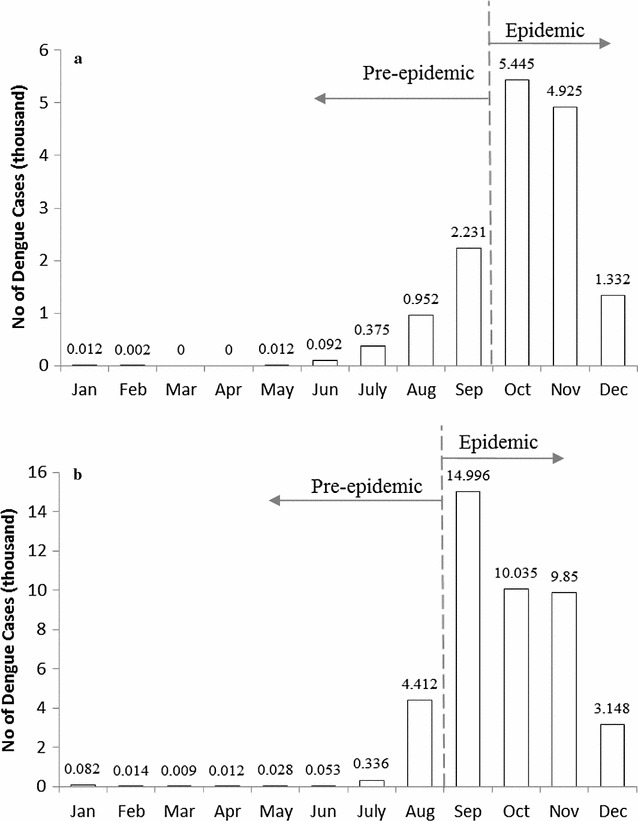
Monthly number of dengue cases in **a** 2014 and **b** 2015. In 2014, the period of Jan.–Sep. is defined as pre-epidemic and Oct.–Dec. as epidemic; in 2015, the period of Jan.–Aug. is defined as pre-epidemic and Sep.–Dec. as epidemic stage

Figure 4 shows the monthly variations of dengue cases in southern Taiwan, 2014 and 2015, and it indicates that there were significant epidemic seasons in these 2 years. We defined the month with the highest dengue cases as the start of epidemic season. Therefore, we categorized the periods of October to December of 2014 and September to December of 2015 as epidemic seasons. We further investigated the association of neighborhood structures and dengue diffusion between pre-epidemic and epidemic seasons in these 2 years.

Table 1 summarizes the population density, number of townships and dengue incidence during the pre-epidemic and epidemic periods in 7 urbanization stratifications, including remote, rural, ageing, general, new-developed, medium-density and high-density areas. It shows that dengue cases were concentrated in medium- and high-density areas in both the pre-epidemic and epidemic stages. Therefore, human mobility between townships with high urbanization levels could be critical routes of spatial transmission.

**Table 1:** Summarized Statistics in urbanization levels

Township stratification	Counts of towns	Avg. population density, person/km^2^	Dengue incidence rate, per 10,000 people	
			Pre-epidemic	Epidemic
Remote area	22	132.9	1.07	5.5
Rural area	18	121.5	0.81	7.24
Aging society area	10	163.1	0.64	4.01
General area	21	616.2	2.52	14.08
Newly developed area	19	1870.8	13.56	72.5
Medium-density urban area	9	5421.5	24.69	116.23
High-density urban area	9	10,746.7	24.97	196.16

### Spatial weights and neighborhood structures

Spatial proximity and human mobility may influence neighborhood diffusion of dengue epidemics. Different definitions of neighborhood structures reflect the effects of spatial interactions. We defined three neighborhood structures, including Queen Contiguity, Distance-threshold weights and matrix of human mobility, for investigating the influence of different types of neighborhood structures on dengue diffusion.

#### Queen contiguity weights

The queen contiguity is one of the standard contiguity-based spatial weighting methods in geographic analysis. It determines neighboring units as those that have any point in common, including both common boundaries and common corners. The spatial weights in a queen contiguity matrix (W_Q_) represent townships that share administration boundaries and have higher possibilities of interacting with each other. Based on queen contiguity, each township has an average of 5.14 neighboring townships in our study.

#### Distance-threshold weights

The distances among townships could influence the extent of daily mobility. The queen contiguity cannot incorporate the effect of distance on spatial interaction. The extent of daily mobility is measured by the journeys someone takes from home to work and back again. The Institute of Transportation identified 20 km as the average daily journey distance for urban trips [39]. Therefore, we measured distances between centroids of townships for establishing a spatial weight matrix (W_D_) that defined the townships within 20 km as the criteria of a neighborhood for modeling spatial interactions. In other words, the distance-threshold weights can reflect spatial interactions between townships within 20 km.

#### Matrix of human mobility

**Fig. 5:**
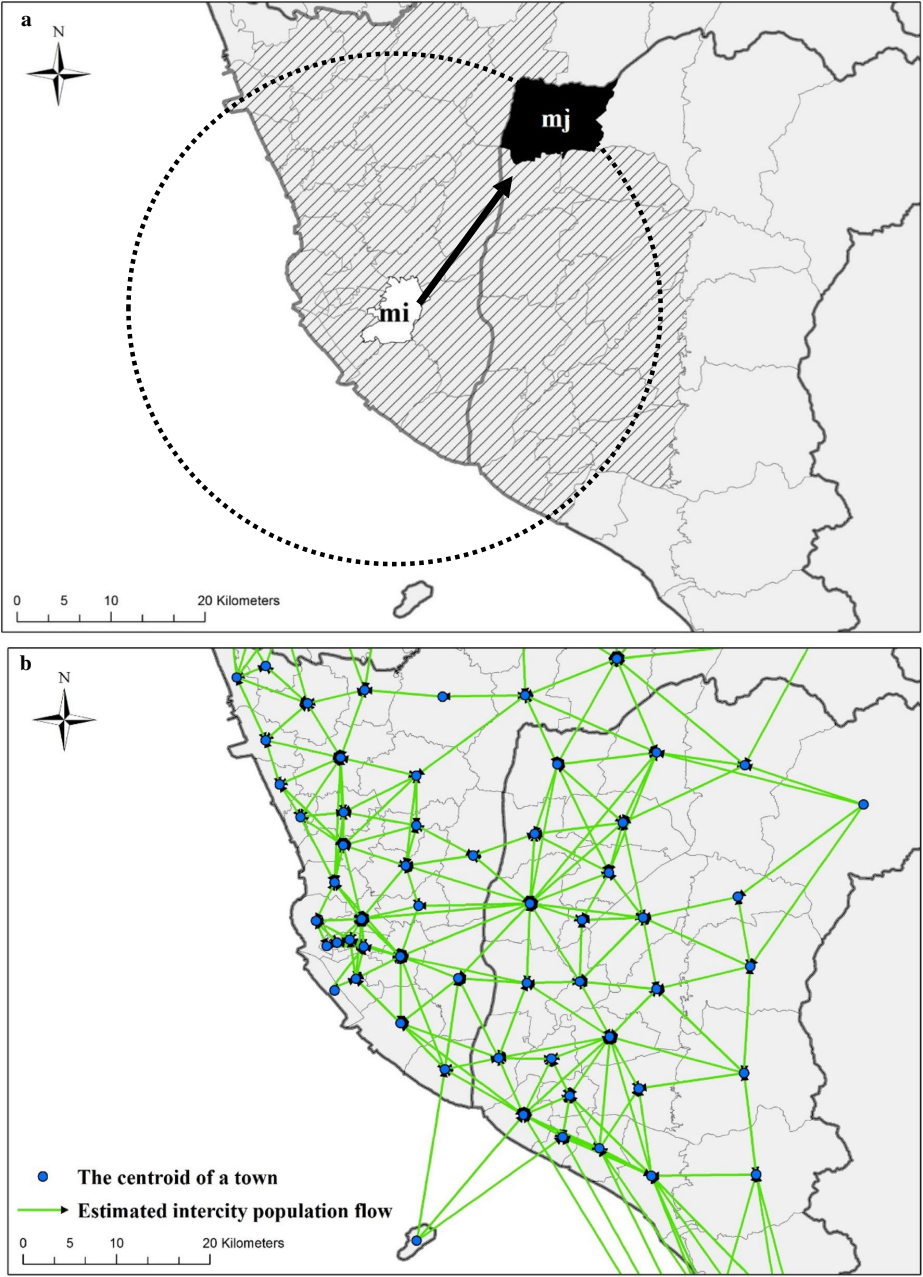
Illustrations of **a** an example of using a radiation mode to estimate population flow from m_i_ (source) to m_j_ (destination), which considers total population in the circle centered at m_i_ and touching m_j_ excluding the source (m_i_) and the destination (m_j_) population. More population in the circle represent people in the mi have more attractive (e.g. opportunity of jobs), and it decreases mobility propensity from mi to m_j_; **b** network connectivity structure of human mobility estimated by a radiation model to represent spatial interaction

The weights of Queen Contiguity and Distance-threshold reflect geometric characteristics of neighborhood structures rather than the patterns of human mobility. In other words, these definitions cannot differentiate the directions and volumes of population flow between urban-to-urban and urban-to rural areas. Therefore, we adopted the concept of radiation model proposed by Simini et al. [32] for quantifying spatial interaction between townships (Eq. 1). The radiation model can estimate routine human mobility, which reflects daily commute [32]. Therefore, we used the proportions of commuters from one to another location as the spatial weight matrix for quantifying spatial associations among locations.

1 $$T_{ij} = m_{i} \frac{{m_{j} }}{{\left( {m_{i} + s_{ij} } \right)\left( {m_{i} + m_{j} + s_{ij} } \right)}}$$

where T_ij_ is the proportion of the commuters in township i travelling to township j; mi and m_j_ are the populations in townships i and j, respectively; and s_ij_ is the total population in the circle centered at i and touching j, excluding the source and destination population as shown Fig. 5a. The population in the circle (s_ij_) represents attraction (e.g., opportunity of jobs) to mi. If s_ij_ is larger, it indicates that the population in the m_i_ has more mobility alternatives, which decreases the mobility propensity from m_i_ to m_j_. The parameter-free model is validated in various behaviors of human mobility, including journeys with a short travel time, daily commute, and migration [32]. We used the model to estimate the trips for constructing an Original-Destination Matrix W, W(i,j) is the estimated trips from township i to j and the transpose matrix W^T^(i,j) is then the estimated trips from j to i. Therefore, we generated a fully connected symmetric matrix W_F_ = W + W^T^, which can capture the spatial interactions between townships, and W_F_ is the spatial weights we used to measure the human mobility between townships (Fig. 5b). Township population statistical data for the radiation model is from the Department of Household Registration, Ministry of the Interior in Taiwan.

**Fig. 6:**
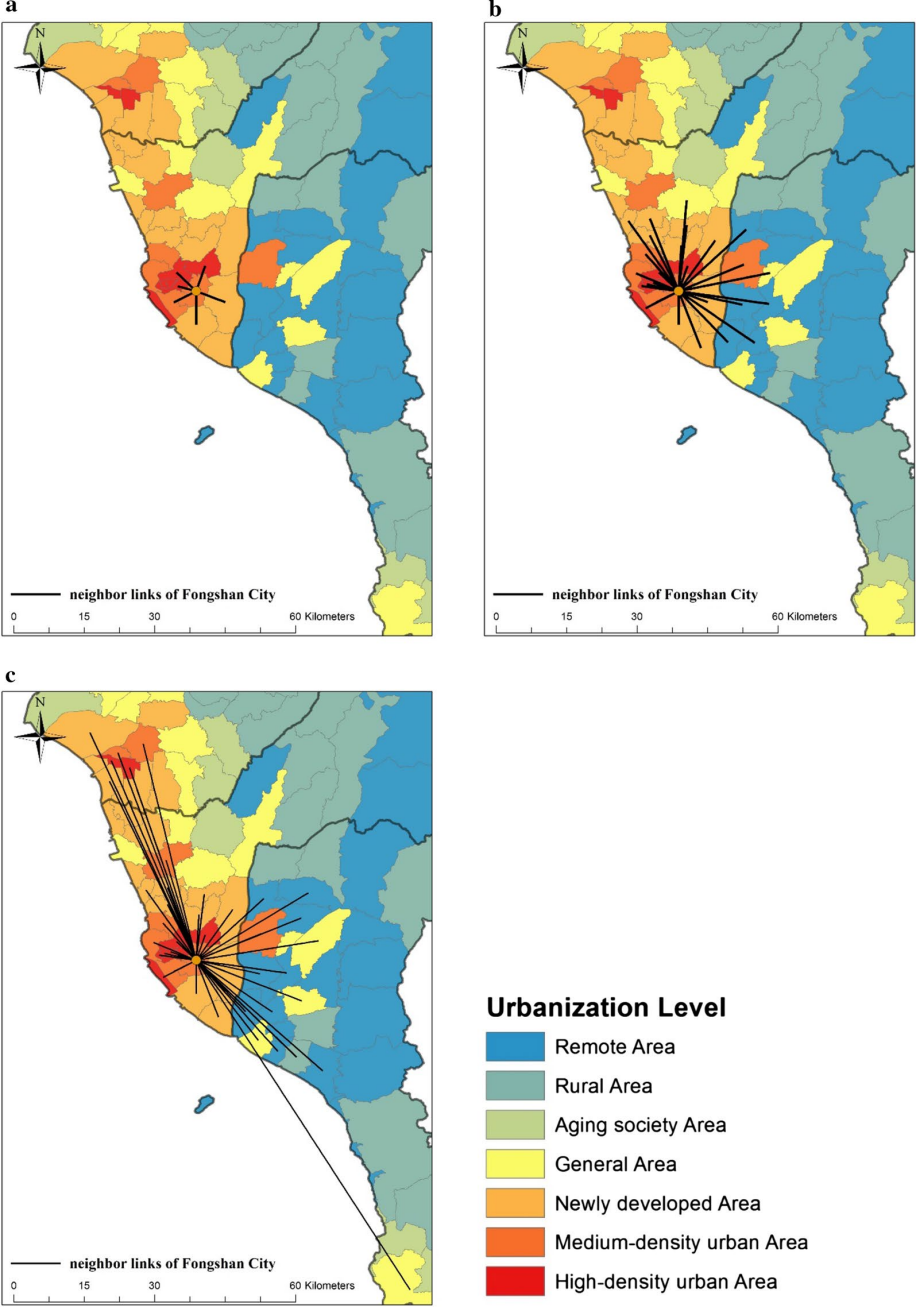
An example of neighborhood structures of Fongshan District of Kaohsiung City based on three definitions: **a** queen contiguity, **b** distance-threshold weights and **c** structure of human mobility

Figure 6 illustrates an example of the neighborhood structures of Fongshan District in Kaohsiung City based on these three criteria. The neighborhood structures in Fig. 6a, b reflect the geometric characteristics of the administration boundary, and the spatial interactions in Fig. 6c capture the spatial variations of human mobility and characteristics of urbanization.

### Statistical analysis

We used spatial regression modeling for investigating the neighborhood effects on spatial–time diffusion of dengue incidence between a township and its neighboring area. Spatial lag model, one of spatial regression specifications, adds a spatial lag operator to the outcome variable (e.g. disease incidence) for investigating neighboring effects as diffusion process [40, 41]. Therefore, by integrating dengue cases in different periods, in this study, spatial lag model was used for quantitatively measuring dengue diffusion effects in different periods.

We developed three statistical model specifications: Model 1 only measures the neighboring effect of the pre-epidemic period (t1), and Model 2 considers the diffusion effect of dengue incidence in neighboring townships during both the pre-epidemic (t1) and epidemic (t2) periods. Comparing these two models can differentiate diffusion effects from the pre-epidemic (t1) and epidemic (t2) periods to investigate the influence of neighborhood structures on epidemic progression. Model 3 considers the second-order neighboring townships of the pre-epidemic period (t1) for quantifying the relatively long-distance diffusion effect during the period.

Moreover, different settings of neighborhood structures, including Queen Contiguity, distance-threshold weights and structure of human mobility, are compared in each model for each year so that we could systematically understand which neighborhood structure is more appropriate for the discussion on spatial autocorrelation of dengue and the result we found would be more convinced. Models 1 was fitted to data using ordinary least squares (OLS) method and Models 2 and 3 were fitted using maximum likelihood estimation (MLE) with the R package spdep. The Akaike information criterion (AIC) is used as the performance indicator of model fitting. A model with a lower AIC value has a better explanation for dengue diffusion.

**Fig. 7:**
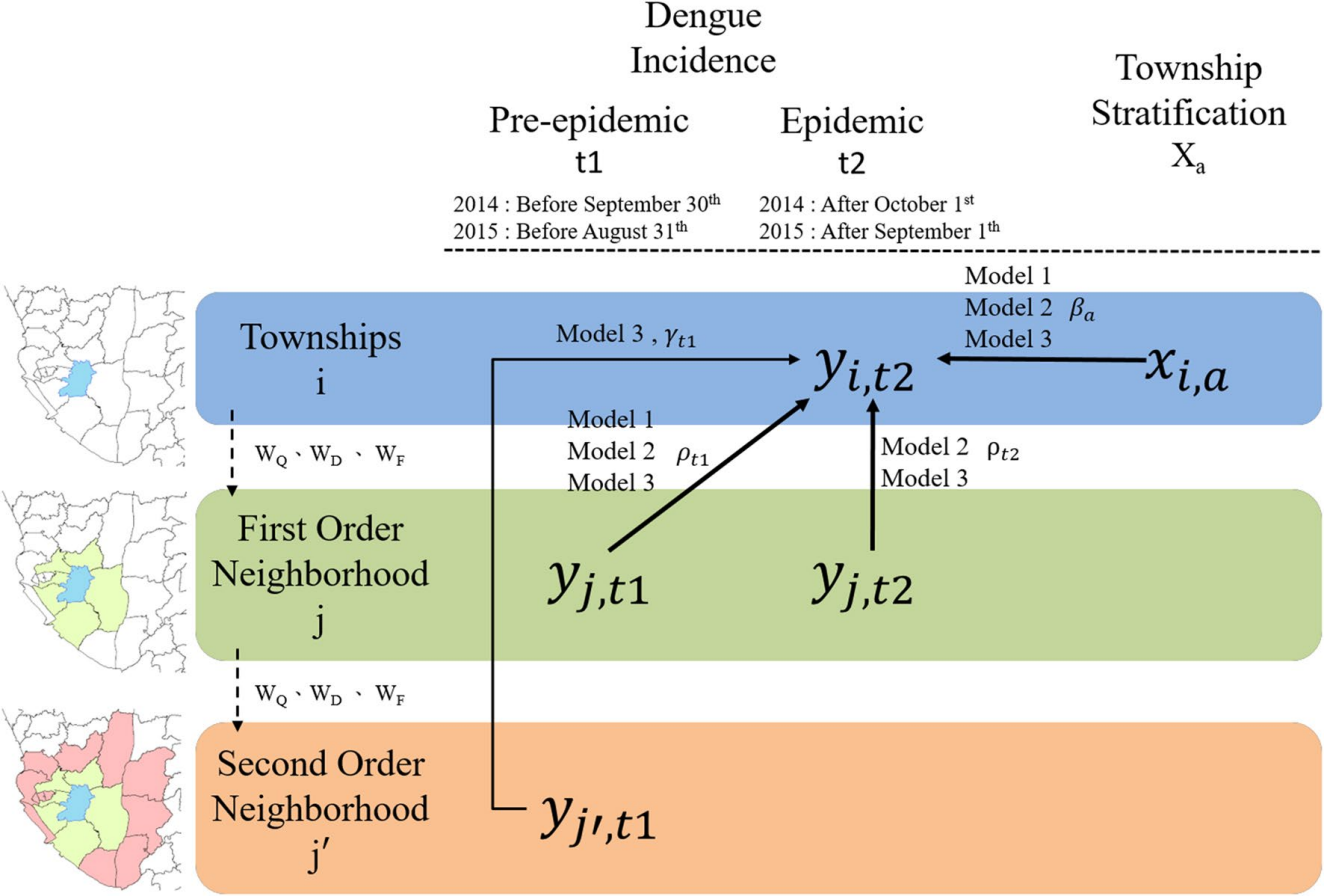
Study framework of statistical analysis: Different colors represent different-order neighborhood structures. The variables in the first layer (blue) represent a township i; the second layer (green) represent the 1-order neighborhood (immediate neighbors), and the third layer (pink) represent the 2-order neighborhood (distant neighbors). The arrows represents influence relations between these variables

The model framework of statistical analysis is shown in Fig. 7. Different colors of the layers represent different-order neighborhood structures. The variables in the first layer (blue) represent a township i; the second layer (green) represent the 1-order neighborhood (immediate neighbors), and the third layer (pink) represent the 2-order neighborhood (distant neighbors). The arrows represents influence relations between these variables. Detailed model specifications are described as follows.

#### Model 1: pre-epidemic neighborhood effect model

We used ordinary least squares (OLS) Regression to investigate the diffusion effect of dengue incidence in neighboring townships during the pre-epidemic period, controlling for urbanization levels, as shown in Eq. 2

2 $${\text{y}}_{t2} = \mathop \sum \limits_{a = 1}^{6} \beta_{a} x_{a} + \rho_{t1} W{\text{y}}_{t1} +\upvarepsilon$$

where y_t2_ is the logarithmic dengue incidence of a township during the epidemic period (t2) and x is the urbanization level, which is a categorical variable. There are six dummy variables used to capture seven urbanization levels. *β*_a_ is the marginal effect for one urbanization type (a). W is a spatial weight matrix, including the above-mentioned W_Q_, W_D_ and W_F_, for investigating the neighborhood structures. $Wy_{t1}$ measures dengue incidence in pre-epidemic period (t1) and its coefficient *ρ*_*t*1_ is the marginal neighboring effect during the pre-epidemic period (t1), which can capture diffusion process of dengue epidemics from t1 to t2. ɛ is the regression residual.

#### Model 2: current neighborhood effect model

We used spatial lag models (SLM) to investigate the diffusion effect of dengue incidence in neighboring townships during both the pre-epidemic (t1) and epidemic (t2) periods, controlling for urbanization levels, as shown in Eq. 3.

3 $${\text{y}}_{t2} = \rho_{t2} {\text{W}}y_{t2} + \mathop \sum \limits_{a = 1}^{6} \beta_{a} x_{a} + \rho_{t1} Wy_{t1} +\upvarepsilon$$

where *β*_a_ is the marginal effect for one urbanization type (a). W is a spatial weight matrix, including the above-mentioned W_Q_, W_D_ and W_F_, for investigating the neighborhood structures. Similar as Model 1, $\rho_{t2} {\text{W}}y_{t2}$ measures diffusion effect of dengue incidence in epidemic period (t2). ρ_t2_ is the marginal neighboring effect during the epidemic period (t2), and ρ_t1_ is the effect during the pre-epidemic period (t1), and ɛ is the regression residual.

#### Model 3: long distance model

We used spatial Durbin models (SDM) to investigate the 2-order neighborhood effect in the pre-epidemic period as shown in Eq. 4. The 2-order neighborhood for a township refers to the neighbors of neighboring townships. In other words, the 2-order neighboring effect can capture the effect of relatively long-distance diffusion. Model 3 is the same as Model 2, except for the term $\gamma_{t1} W\left( {Wy_{t1} } \right)$, which captures 2-order neighboring townships.

4 $$y_{t2} = \rho_{t2} {\text{W}}y_{t2} + \mathop \sum \limits_{a = 1}^{6} \beta_{a} x_{a} + \rho_{t1} Wy_{t1} + \gamma_{t1} W\left( {Wy_{t1} } \right) +\upvarepsilon$$

where *β*_a_ is the marginal effect for one urbanization type (a). W is a spatial weight matrix, including the above-mentioned W_Q_, W_D_ and W_F_, for investigating the neighborhood structures. ρ_t2_ is the marginal neighboring effect during the epidemic period (t2); ρ_t1_ is the effect during the pre-epidemic period (t1); γ_t1_ is the marginal 2-order neighboring effect during the pre-epidemic period (t1); and ɛ is the regression residual.

## Results

Tables 2 and 3 summarize the effects of different model configurations and epidemic progression on dengue incidence in 2014 and 2015.

**Table 2:** Model results of 2014 dengue epidemic using the matrix of population mobility as spatial weights

Independent variables	Model 1	Model 2	Model 3
Intercept	1.527(0.79)	1.149(0.69)	3.039***(0.75)
Spatial-lag of dengue incidence			
1st order neighbors in pre-epidemic period *ρ*_*t*1_	1.107***(0.08)	0.671***(0.11)	− 0.002(0.17)
1st order neighbors in epidemic period *ρ*_*t*2_	–	0.428***(0.09)	0.306**(0.10)
2nd order neighbors in pre-epidemic period *γ*_*t*1_	–	–	1.001***(0.20)
Urbanization levels^a^			
Rural area *β*_1_	− 0.229(0.24)	− 0.099(0.21)	0.015(0.198)
Aging society area *β*_2_	0.641*(0.31)	0.569*(0.27)	0.384(0.25)
General area *β*_3_	0.236(0.24)	0.26(0.21)	0.465*(0.20)
Newly developed area *β*_4_	0.964***(0.24)	0.647**(0.22)	0.771***(0.20)
Medium-density urban area *β*_5_	0.791*(0.30)	0.595*(0.27)	0.639*(0.24)
High-density urban area *β*_6_	0.521(0.33)	0.313(0.29)	0.456(0.27)
Performance of model fitting			
AIC	259.54	247.11	226.01
R-square	0.72	–	–

The value in parentheses is standard error

*p value < 0.05; **p value < 0.01; ***p value < 0.001

^a^“Remote area” as reference category

**Table 3:** Model results of 2015 dengue epidemic using the matrix of population mobility as spatial weights

Independent variables	Model 1	Model 2	Model 3
Intercept	− 3.08***(0.90)	− 1.56(0.81)	− 0.53(0.92)
Spatial-lag of dengue incidence			
1st order neighbors in pre-epidemic period *ρt*1	0.50***(0.09)	0.27*(0.08)	− 0.007(0.16)
1st order neighbors in epidemic period *ρt*2	–	0.47***(0.08)	0.467***(0.08)
2nd order neighbors in pre-epidemic period *γt*1	–	–	0.40*(0.19)
Urbanization levels^a^			
Rural area *β*_1_	− 0.11(0.25)	− 0.10(0.21)	− 0.14(0.20)
Aging society area *β*_2_	− 0.37(0.30)	− 0.45(0.25)	− 0.53(0.25)
General area *β*_3_	0.42(0.24)	0.40(0.21)	0.33(0.20)
Newly developed area *β*_4_	1.34***(0.26)	0.77**(0.24)	0.66*(0.24)
Medium-density urban area *β*_5_	1.94***(0.32)	1.31***(0.29)	1.21***(0.29)
High-density urban area *β*_6_	2.47***(0.33)	1.44***(0.33)	1.36***(0.32)
Performance of model fitting			
AIC	259.25	239.52	237.17
R-square	0.708	–	–

The value in parentheses is standard error

*p value < 0.05; **p value < 0.01; ***p value < 0.001

^a^“Remote area” as reference category

Our findings show consistent results for the dengue epidemics of 2014 and 2015.

Table 4 summarized the model-fitting performances (AIC values) for different settings of neighborhood structures and spatial model configurations for dengue diffusion in 2014 and 2015. It shows that Model 3 (long distance model) with structure of human mobility has the lowest AIC value. (Detailed statistical results for all of the models can be found in Additional file 1: Tables S1–S4). This finding indicates that population mobility as the neighborhood structure can better explain the relatively long-distance (2nd-order) and immediate (1st-order) neighboring dengue diffusion in different periods.

**Table 4:** AIC values for different settings of neighborhood structures and spatial model configurations for dengue diffusion

Neighborhood structures	2014			2015		
	Model 1	Model 2	Model 3	Model 1	Model 2	Model 3
Queen contiguity	328.4	319.7	320.6	278.0	278.1	275.7
Distance-threshold weights	256.0	254.3	255.0	270.7	268.0	269.9
Structure of human mobility	259.5	247.1	226.0	259.3	239.5	237.2

Model 1: pre-epidemic neighborhood effect model, Model 2: current neighborhood effect model, and Model 3: long distance model

Regarding urbanization levels in the models with population mobility structures, in Tables 2 and 3, Model 3 shows areas that are newly developed, medium-density and high-density are associated with significantly higher dengue incidence relative to remote areas. Interestingly, the results of Model 3 also indicate that the dengue incidence during the epidemic period is significantly associated with 1st-order neighbors during the epidemic period (t2) and 2nd-order neighbors which are relatively distant townships compare with 1st-order neighbors during the pre-epidemic period (t1) since the coefficient of *ρ*_*t*2_ and *γ*_*t*1_ in Model 3 are significant. Our results show the spatial–temporal hierarchy of dengue diffusion: the dengue incidence in a township would be impacted by immediate neighbors during pre-epidemic and epidemic periods, and also with more distant neighbors (based on mobility) in pre-epidemic periods.

## Discussion

Recent literature has indicated that one of the major driving forces of geographic expansion of dengue is human mobility on different scales, including within-city, intercity in a region, and international travels [13, 15, 16]. To quantify the effects of mobility on the spatial diffusion of dengue epidemics, previous literature replaced spatial mobility structures with geometric relationships in spatial regression models [4, 25]. However, these geometric relationships cannot fully reflect realistic spatial interactions [27, 28]. On the other hand, large-scale intercity individual mobility routes are difficult to collect, track, and access in most countries. Therefore, our study proposed a framework for model configuration to profile intercity dengue diffusion. First, we used a radiation model to construct the structure of human mobility as neighborhood weight in the spatial regression model. Second, we categorized the pre-epidemic and epidemic periods for investigating the time-lag effect on dengue diffusion. Lastly, we incorporated 2nd-order and 1st-order neighboring structures in our model to quantify relatively long-distance and immediate diffusion effects. Compared with conventional model configuration in spatial regression analysis, our proposed radiation model specification demonstrates better model fitting performance in both 2014 and 2015, which indicates that the structure of human mobility has better explanatory power in dengue diffusion than geometric relationships. The model results in the 2014 and 2015 dengue epidemics have consistent findings, indicating that intercity mobility and urbanization could be driving forces of large-scale epidemic expansion of dengue [12, 14, 17, 19].

Our findings show that highly-urbanized areas are positively associated with dengue incidence in 2014 and 2015, which is consistent with the literature, such as in [3, 4, 42]. In southern Taiwan, household plant plotting in townhouse buildings and small-area flooding water in yards after an extensive rainfall provide appropriate habitats for dengue vector mosquitoes in urbanized areas. Meanwhile, areas with high population densities could help mosquitoes bite people more easily [4]. On the other hand, female *Aedes aegypti*, a major dengue vector mosquito, are most active during the daytime, which means that there is a high frequency of biting by *A. aegypti* for people gathering in public places, which increases the risk of dengue outbreak [43–45].

Regarding geographic expansion of epidemics, our study identifies a significant neighboring diffusion effect on dengue epidemics. The dengue incidence in a township would be impacted by neighboring townships during either the pre-epidemic or epidemic period. This finding implies that the potential sources of diffusion for the township might be its neighboring townships with high dengue incidence. The result reflects the structure of human mobility as spatial interactions causing epidemic expansion [4, 25]. Moreover, the 2nd-order neighboring structure also has a significant effect on the township during the pre-epidemic period, which reflects the relatively long-distance diffusion effect. In other words, our results demonstrate a “ripple” process of dengue diffusion, which means that the immediate (first-order) neighboring effect occurs in the initial epidemic wave and that the wider geographic expansion occurs in a later epidemic wave, which is affected by the distant (second-order) neighboring effect.

Cliff et al. [46] categorized spatial diffusion patterns into three major types. Contagion results from direct contact for spreading. Relocation describes diffusion source shifts to another distant location. Hierarchy refers to transmission through an ordered sequence of settlements rather than following a distance-based neighborhood structure. Numerous studies have interpreted the epidemiological implications of these diffusion patterns [47–49]. In most cases, the structure of epidemic diffusion is often a mixture of these patterns [13, 49, 50], and relocation and hierarchical patterns cause long-distance dispersion [51]. Our proposed model has profiled possible mechanisms for these patterns. Conventional geometric relationships, such as contiguity-based and distance-threshold weighting schemes, are based on a distance-decayed structure, which could capture the characteristics of contagious diffusion. However, geometric relationships do not reflect topographic variability and long-distance interactions due to transportation. A radiation model considered in our study captures the structure of intercity interactions, reflecting patterns of human mobility. For example, in Fig. 6c, population flows from Fongshan city (high-density urbanized areas) are not only to neighboring townships, but there are also flows to other distant high-density cities in Tainan. This means that our model can capture realistic urban-to-urban interactions partially, which could cause relocation and hierarchical diffusion. Brockmann and Helbing [23] also proposed a concept of “effective distance,” which replaces conventional geographic distance with the matrix of passenger flux through air traffic between cites for predicting global disease arrival times. In summary, large-scale geographic expansion of epidemic propagation is difficult to explain only by the geometric structure of administration boundaries or geographic distances of centroids between cities. Population mobility or passenger flows could more reasonably capture the structure of spatial interactions and long-distance diffusion patterns. In other words, hub cities could play a role as a “bridge” for large-scale transmission and make townships connecting to hub cities more vulnerable to dengue epidemics.

The study has some limitations. First, the mobility structure was estimated by a parameter-free radiation model, which only considers the population size of a city rather than the empirical or surveyed mobility data. Although the estimated mobility structure captures urban-to-urban interactions, we did not consider the detailed mobility behaviors of individuals or even dengue patients, such as choices of transportation modes or purposes of the trip. Further investigation on more detailed intercity mobility structure is warranted. Second, in addition to human mobility, dengue diffusion is a complex process in terms of the spatial–temporal variability in mosquito density, effectiveness of control measures, pathogen activity and host immunity [43, 52]. However, most of these factors are not available for intercity-scale studies. It is necessary to develop reliable sampling schemes for collecting these data in further investigations. Thirdly, we only used the most severe epidemics in Taiwan as case study. Although the findings are consistent in both years, it does not imply the findings still valid in other years. It would be worth to incorporate long-term longitudinal epidemic data for investigating the influence of human mobility on dengue diffusion. Our findings may suggest that, in severe epidemic years, human mobility plays a significant role in intercity dengue diffusion. Finally, the spatial heterogeneity of intercity diffusion effect should also be considered in further investigation. For example, a geographic weighted regression (GWR) can be further used to differentiate the effect of human mobility on dengue incidence in each township. Spatially-varying relationships of neighboring effects on dengue incidence could provide the heath authority for implementing better adopt specific control and prevention strategies to specific areas.

## Conclusions

The study proposed a study framework for investigating relatively long-distance and immediate neighboring diffusion effects on epidemic propagation and clarified the role of intercity-scale human mobility structure and urbanization levels as driving forces in large-scale dengue transmission. Our findings suggest that the intercity mobility structure reflects urban-to-urban interactions, which causes a mixture of relocation and hierarchical diffusion patterns for large-scale dengue epidemics in southern Taiwan. This can be identified as a “ripple” process wherein an immediate neighboring effect occurs in the first stage and wider geographic expansion occurs in a later stage, which is influenced by the distant neighboring diffusion effect.

## Additional file

**Additional file 1.** Detailed statistical results for all of the models.

## Abbreviations

AIC: Akaike information criterion
AR5: The Fifth Assessment Report
CDC: Centers of Disease Control
CDRS: call detail records
IPCC: Intergovernmental Panel on Climate Change
GPS: Global Positioning System
GWR: geographically weighted regression
OLS: ordinary least squares
SDM: spatial Durbin model
SLM: spatial lag model
