# Supplementary material for: Evaluating neighborhood structures for modeling intercity diffusion of large-scale dengue epidemics
Source: Int J Health Geogr. 2018 May 3;17:9. doi: 10.1186/s12942-018-0131-2 (PMC5934834; doi:10.1186/s12942-018-0131-2)
Supplement: Supplementary file 1 — Additional file 1. Detailed statistical results for all of the models. [file 12942_2018_131_MOESM1_ESM.docx]

**Additional file**

Table S1: Model results for the 2014 dengue epidemic using the Queen Contiguity as spatial weights

| Independent Variables | Model 1 | Model 2 | Model 3 |
| --- | --- | --- | --- |
| Intercept | -4.07***(0.73) | -4.29***(0.74) | -4.07***(0.74) |
| **spatial-lag of dengue incidence** |  |  |  |
| 1^st^ order neighbors in pre-epidemic period $\rho_{t1,F}$ | 0.519***(0.08) | 0.049(0.08) | -0.301(0.35) |
| 1^st^ order neighbors in epidemic period $\rho_{t2,F}$ | - | 0.472***(0.09) | 0.45***(0.09) |
| 2^nd^ order neighbors in pre-epidemic period $\gamma_{t1,F}$ | - | - | 0.397(0.37) |
| **Urbanization levels** ^a^ |  |  |  |
| Rural Area $\beta_{1,F}$ | 0.008(0.98) | 0.071(0.30) | 0.073(0.30) |
| Aging society Area $\beta_{2,F}$ | 0.006(0.88) | 0.039(0.37) | 0.033(0.37) |
| General Area $\beta_{3,F}$ | 0.127(0.71) | 0.109(0.30) | 0.132(0.30) |
| Newly developed Area $\beta_{4,F}$ | 1.07**(0.002) | 0.644*(0.31) | 0.651*(0.31) |
| Medium-density urban Area $\beta_{5,F}$ | 1.20**(0.005) | 0.877*(0.38) | 0.877*(0.38) |
| High-density urban Area $\beta_{6,F}$ | 1.69***(0.000) | 1.321***(0.39) | 1.372***(0.39) |
| **Performance of model fitting** |  |  |  |
| AIC | 328.41 | 319.67 | 320.56 |
| R-square | 0.47 | - | - |

^a^  “Remote Area” as reference category.

* p-value < 0.05; ** p-value < 0.01; *** p-value < 0.001

Table S2: Model results for the 2014 dengue epidemic using the distance-threshold weights as spatial weights

| Independent Variables | Model 1 | Model 2 | Model 3 |
| --- | --- | --- | --- |
| Intercept | 3.675***(0.93) | 2.986*(0.92) | 3.9**(1.32) |
| **spatial-lag of dengue incidence** |  |  |  |
| 1^st^ order neighbors in pre-epidemic period $\rho_{t1,F}$ | 1.371***(0.10) | 0.989***(0.18) | 0.681*(0.34) |
| 1^st^ order neighbors in epidemic period $\rho_{t2,F}$ | - | 0.329*(0.14) | 0.245(0.174) |
| 2^nd^ order neighbors in pre-epidemic period $\gamma_{t1,F}$ | - | - | 0.493(0.48) |
| **Urbanization levels** ^a^ |  |  |  |
| Rural Area $\beta_{1,F}$ | 0.074(0.24) | 0.12(0.23) | 0.235(0.34) |
| Aging society Area $\beta_{2,F}$ | 0.752*(0.30) | 0.725*(0.28) | 0.801**(0.29) |
| General Area $\beta_{3,F}$ | 0.587*(0.24) | 0.527*(0.23) | 0.619*(0.24) |
| Newly developed Area $\beta_{4,F}$ | 0.943***(0.23) | 0.73**(0.23) | 0.839***(0.25) |
| Medium-density urban Area $\beta_{5,F}$ | 1.006**(0.29) | 0.824**(0.28) | 0.957***(0.30) |
| High-density urban Area $\beta_{6,F}$ | 1.517***(0.30) | 1.295***(0.29) | 1.426***(0.31) |
| **Performance of model fitting** |  |  |  |
| AIC | 256.01 | 254.28 | 255.03 |
| R-square | 0.73 | - | - |

^a^  “Remote Area” as reference category.

* p-value < 0.05; ** p-value < 0.01; *** p-value < 0.001

Table S3: Model results for the 2015 dengue epidemic using the Queen Contiguity as spatial weights

| Independent Variables | Model 1 | Model 2 | Model 3 |
| --- | --- | --- | --- |
| Intercept | -6.11***(0.63) | -5.89***(0.65) | -6.36***(0.66) |
| **spatial-lag of dengue incidence** |  |  |  |
| 1^st^ order neighbors in pre-epidemic period $\rho_{t1,F}$ | 0.18**(0.06) | 0.07(0.08) | 0.56*(0.25) |
| 1^st^ order neighbors in epidemic period $\rho_{t2,F}$ | - | 0.173(0.09) | 0.21(0.10) |
| 2^nd^ order neighbors in pre-epidemic period $\gamma_{t1,F}$ | - | - | -0.57*(0.28) |
| **Urbanization levels** ^a^ |  |  |  |
| Rural Area $\beta_{1,F}$ | 0.16(0.26) | 0.21(0.25) | 0.21(0.25) |
| Aging society Area $\beta_{2,F}$ | -0.02(0.31) | 0.01(0.30) | 0.09(0.30) |
| General Area $\beta_{3,F}$ | 0.76**(0.25) | 0.77**(0.24) | 0.81**(0.23) |
| Newly developed Area $\beta_{4,F}$ | 1.79***(0.26) | 1.62***(0.27) | 1.61***(0.26) |
| Medium-density urban Area $\beta_{5,F}$ | 2.41***(0.33) | 2.21***(0.33) | 2.19***(0.33) |
| High-density urban Area $\beta_{6,F}$ | 3.03***(0.34) | 2.70***(0.36) | 2.59***(0.35) |
| **Performance of model fitting** |  |  |  |
| AIC | 277.96 | 278.11 | 275.74 |
| R-square | 0.653 | - | - |

^a^  “Remote Area” as reference category.

* p-value < 0.05; ** p-value < 0.01; *** p-value < 0.001

Table S4: Model results for the 2015 dengue epidemic using the distance-threshold weights as spatial weights

| Independent Variables | Model 1 | Model 2 | Model 3 |
| --- | --- | --- | --- |
| Intercept | -2.81*(1.28) | -1.75(1.31) | -1.49(1.44) |
| **spatial-lag of dengue incidence** |  |  |  |
| 1^st^ order neighbors in pre-epidemic period $\rho_{t1,F}$ | 0.53***(0.13) | 0.39**(0.13) | 0.26(0.35) |
| 1^st^ order neighbors in epidemic period $\rho_{t2,F}$ | - | 0.31*(0.13) | 0.32*(0.13) |
| 2^nd^ order neighbors in pre-epidemic period $\gamma_{t1,F}$ | - | - | 0.15(0.41) |
| **Urbanization levels** ^a^ |  |  |  |
| Rural Area $\beta_{1,F}$ | -0.11(0.26) | -0.12(0.25) | -0.13(0.25) |
| Aging society Area $\beta_{2,F}$ | -0.44(0.32) | -0.45(0.30) | -0.48(0.31) |
| General Area $\beta_{3,F}$ | 0.30(0.27) | 0.23(0.26) | 0.21(0.26) |
| Newly developed Area $\beta_{4,F}$ | 1.34***(0.29) | 0.97**(0.31) | 0.96**(0.31) |
| Medium-density urban Area $\beta_{5,F}$ | 2.08***(0.33) | 1.76***(0.35) | 1.74***(0.35) |
| High-density urban Area $\beta_{6,F}$ | 2.81***(0.33) | 2.34***(0.36) | 2.37***(0.36) |
| **Performance of model fitting** |  |  |  |
| AIC | 270.69 | 268 | 269.87 |
| R-square | 0.676 | - | - |

^a^  “Remote Area” as reference category.

* p-value < 0.05; ** p-value < 0.01; *** p-value < 0.001
